# 生物信息学方法对影响肺癌发生发展关键基因的初步筛选

**DOI:** 10.3779/j.issn.1009-3419.2012.11.07

**Published:** 2012-11-20

**Authors:** 俊龙 王, 俊杰 石, 文洲 刘, 宇 孙, 华富 周

**Affiliations:** 530021 南宁，广西医科大学第一附属医院心胸外科 Department of Cardiothoracic Surgery, the First Affiliated Hospital, Guangxi Medical University, Nanning 530021, China

**Keywords:** 肺肿瘤, 基因富集, *Meta*分析, 关键基因, 紧密连接通路, Lung neoplasms, Gene set enrich analysis, *Meta*-analysis, Crucial gene, Tight junction

## Abstract

**背景与目的:**

肺癌是最常见的恶性肿瘤之一。对于肺癌基因芯片的研究已在很多报道里提及，但是鲜有报道汇总所有的基因芯片数据来研究肺癌的共同表达通路从而挖掘出特殊的基因标记来作为治疗肺癌的靶点。本研究从肺癌相关的基因芯片数据库（GEO DataSets）中初步筛选出与肺癌发生发展有关键联系的基因与通路。

**方法:**

运用基因富集（gene set enrichment analysis, GSEA）等生物信息学方法比较选择出的6套肺癌基因芯片表达谱数据，初步筛选出在转录水平上影响肺癌的通路及基因。

**结果:**

用GSEA方法分析6组芯片集中所得的通路对比，上调中皆有的通路有3条；下调中皆有的通路有26条。本研究挑选共同存在于6套芯片数据集的下调通路即紧密连接（tight junction）通路里的基因进行*meta*分析，可得差异有统计学意义（*P* < 0.05）的基因11个。

**结论:**

紧密连接通路在肺癌发生发展过程中的共性可能有一定研究意义，可在后续研究中探讨通路里的显著性基因。

## Abstract (en)

**Background and objective:**

Lung cancer is the most common cancer in the world. The gene expression profiling of lung cancer has been extensively investigated. However, only a few studies have identified the possible pathways and significant genes related to lung cancer. The aim of this study is to explore the large number of lung cancer-related microarray datasets and identify the crucial genes that can benefit the understanding of the progression and development of this disease.

**Methods:**

To identify the genes that effected lung cancer at the mRNA level, gene set enrichment analysis was used to analyze six selected gene expression datasets.

**Results:**

Among the six gene expression datasets, 3 up-regulated and 26 down-regulated pathways were found by gene set enrichment analysis. We found 11 significant genes with *P* < 0.05 from the results of tight junction *meta*-analysis of the six data sets.

**Conclusion:**

The tight junction pathway plays an important role in the study of the occurrence and development of lung cancer. Significant genes within the pathways will be further discussed in future studies.

肺癌是最常见的恶性肿瘤之一，对肺癌的早期诊断和有效治疗是全球亟需解决的一个难题。在肿瘤发生发展过程中，有大量伴随基因参与癌基因扩增过程，但是它们并不是我们所要找的关键基因。我们考虑通过探索不同组织类型、不同人群来源的肺癌组织基因芯片，得到芯片结果共同改变的部分，有可能筛选出影响肺癌发生发展的关键基因。目前已有研究通过分析基因表达芯片来挖掘影响肺癌发生发展的通路及基因^[1]^，已识别出大量差异表达的基因。但这些差异基因并没有进行进一步讨论，各个基因芯片的分析结果存在很多不一致性。Mootha等^[2]^提出基因组富集（gene set enrichment analysis, GSEA）分析，该方法能在病例对照类型数据中，基于基因组系统水平上来挖掘影响疾病的基因通路。*Meta*分析可对同一个问题所发表相关研究报告的结果进行收集、统计上的整合，以期获得更准确或更多的结果。Rhodes等^[3]^首先将*meta*分析引入基因芯片数据分析领域。本研究采用GSEA等生物信息学方法对6套^[4-7]^肺癌全基因组表达芯片数据进行研究，挖掘出隐藏在芯片数据下的生物学信息，筛选出影响肺癌发生发展的关键基因，为对肺癌靶向治疗的研究奠定基础。

## 1 材料与方法

### 1.1 研究样本

肺癌有关的基因芯片数据均来源于互联网开放的免费数据库：GEO数据库http://www.ncbi.nlm.nih.gov/geo/中下载。肺癌芯片信息收集在GEO DataSets中以：“lung cancer, homo sapiens”为关键词检索所有公开上传的芯片数据。符合以下标准的数据集将纳入我们的研究中：①所选数据集必须是全基因组的表达mRNA芯片数据；②这些数据是关于肺癌患者和正常对照；③本研究均考虑经标准化或者原始数据集；④所选数据集必须包括超过3个样本以上。最后，有6套芯片数据集纳入我们的研究中（表 1）。

**1:** 6套全基因组数据集的基本情况 Characteristics of datasets selected in the studies

GEO accession	First author or contributor	Chip	Experimental design	Probes	Number of disease	Number of normal
GSE10072	Landi MT^[4]^	Hgu133a	Unpaired	22 k	58	49
GSE18842	Sanchez PA ^[5]^	Hgu133plus2	Unpaired	54 k	46	45
GSE31548	Girard L	Hgu133b	Unpaired	22 k	30	20
GSE31547	Girard L	Hgu133a	Unpaired	22 k	30	20
GSE3268	Wachi S^[6]^	Hgu133a	Paired	22 k	5	5
GSE19804	Lu TP^[7]^	Hgu133plus2	Paired	54 k	60	60
Paired: compare lung tissues from the same patients with Lung cancer tissue; Unpaired: compare lung tissues from the normal people without Lung cancer.						

### 1.2 数据处理

符合我们制定标准的芯片数据，在GEO中下载基因芯片的CEL数据压缩包；若该芯片未提供CEL数据包下载，则下载该数据集的TXT格式的原始数据。通过R语言的Bioconductor 2.10.1版本来对芯片数据进行标准化处理，用软件包affty中的RMA算法对affymetrix平台的原始数据进行背景校正、标准化和log2转换。对每一套数据中每个探针的检验采用成组*t*检验。最后只选取在KEGG中存在的基因进行GSEA的分析。剔除变异四分位距 < 0.5的基因。如果一个基因对应几个探针，我们只保留变异IQR最高的探针。

GSEA通过Bioconductor的Category包进行。只有超过10个基因的类保留，通过*t*检验对每一个通路中的基因进行检验。通过1, 000次循环的排列组合（permutation）获得每个显著通路的*P*值^[1]^。

将得到的6套数据各自上调下调的通路进行总和比较，发现紧密连接通路在6套数据中都表现为下调。我们将每套数据里这条通路的所含基因进行*meta*分析。运用SAS 9.13软件，通过*t*检验把每套数据里紧密连接通路里的每个探针算出*P*值，再通过下列公式算出每个基因的χ^2^值^[8]^。

$
{{\rm{ \mathsf{ χ} }}^2} =  - 2\sum\limits_{{\rm{i}} = 1}^{\rm{K}} {{{\log }_{\rm{e}}}{{\rm{p}}^{\rm{i}}}} 
$

自由度为数据集K的2倍，最后保留*P* < 0.05的基因。对这些基因通路的分析通过DAVID（http://david.abcc.ncifcrf.gov/）中的KEGG库进行分析。

## 2 结果

### 2.1 GSEA分析结果

通过GSEA方法对6套数据集进行功能基因富集，分别找出影响这几个数据集的主要上调通路和下调通路。

GSE10072数据集富集出上调通路50条，下调通路86条。GSE18842富集出61条上调通路；78条下调通路。GSE31548数据集富集出上调通路10条，下调通路54条。GSE31547数据集富集出上调通路40条，下调通路79条。GSE3268数据集富集出上调通路39条，下调通路77条。GSE19804数据集富集出上调通路45条，下调通路87条。

6组数据中所得通路对比，下调通路重叠性较高，共28条（表 2）；上调中皆有的通路为氨基酰-tRNA生物合成aminoacyl-tRNA biosynthesis（属于基因信息分类）；嘧啶代谢pyrimidine metabolism（属于代谢类）；生物碱类合成biosynthesis of alkaloids derived from histidine and purine（属于代谢类）。

**2:** 6组数据集中重叠的28条下调通路 The overlapping 28 down pathways in the studies

Name of the pathways	Name of the pathways
MAPK signaling pathway	Jak-STAT signaling pathway
Calcium signaling pathway	Hematopoietic cell lineage
Cytokine-cytokine receptor interaction	Natural killer cell mediated cytotoxicity
Chemokine signaling pathway	T cell receptor signaling pathway
Phosphatidylinositol signaling system	B cell receptor signaling pathway
Neuroactive ligand-receptor interaction	Fc epsilon RI signaling pathway
Endocytosis	Fc gamma R-mediated phagocytosis
Apoptosis	Leukocyte transendothelial migration
Vascular smooth muscle contraction	Long-term potentiation
TGF-beta signaling pathway	Long-term depression
VEGF signaling pathway	Regulation of actin cytoskeleton
Cell adhesion molecules (CAMs)	GnRH signaling pathway
Tight junction	Melanogenesis
Hypertrophic cardiomyopathy (HCM)	Acute myeloid leukemia

### 2.2 *Meta*分析结果

紧密连接通路属于细胞通讯分类，我们重点研究此条通路。通过R命令语言，得到6组数据集里紧密连接通路各自所含基因探针号。将探针号传至http://david.abcc.ncifcrf.gov/conversion.jsp网站上进行官方名称转换，得到6组数据里该通路所含的基因名称。GSE10072里在紧密连接通路所含差异基因69个，GSE18842含93个，GSE19804含87个，GSE31547含71个，GSE31548含144个，GSE3268含141个。通过上步*Meta*运行结果可得紧密连接通路里差异有统计学意义（*P* < 0.05）的基因11个，它们的名称、*P*值见表 3。筛查这11个基因，其中部分基因与肺癌表达关系密切。

**3:** 共同存在于6套数据集紧密连接通路里的差异显著基因*meta*分析结果 Significance genes of tight junction in *meta* analysis for six datasets

Gene	*P*	Gene	*P*	Gene	*P*	Gene	*P*
*PRKCB*	1.11E-16	*TJP2*	4.78E-09	*CTNNB1*	6.43E-07	*F11r*	0.000, 61
*Jam3*	2.10E-14	*PTENP1*	3.44E-07	*NRAS*	4.20E-05	*csdA*	0.000, 43
*CASK*	5.62E-13	*PTEN*	3.44E-07	*CSDAP1*	0.000, 38		

## 3 讨论

20世纪90年代涌出的基因芯片技术是在固相支持物表面集成大量的分子探针，与标记好的样品杂交然后进行检测分析，能够在同一时间内分析大量基因的表达情况，是一种高效、快速地筛选及检测分析基因活性的新方法，此方法的出现对我们寻找肺癌标记物有重要意义。人们发现单纯的分析基因表达芯片所得数据并不理想，主要因为生物调控网络非常复杂，许多基因不仅局限于发挥一项生物学功能。把基因表达的数据与其功能或已知的信号通路联系起来，才能更好地解释芯片数据，发现基因表达变化的潜在机制。在代谢进程中细胞中的一部分基因经常共同变化，研究这部分共性的基因组成的通路可能比研究单个基因更有意义。因为实验平台、样本、标化方法、分析方法等问题的存在，不同实验室的芯片数据有很多的差异。在众多差异存在的情况下所获得的共同通路可能是在癌症发生发展过程中未经改变的原始部分^[9]^。这部分基因对于我们阐明肺癌的发病机制可能更有意义。

我们选择在6套基因芯片数据中都共同存在下调的紧密连接通路进行研究，这条通路属于细胞通讯分类，比其它代谢类的通路更有研究价值。有研究^[11]^表明紧密连接通路在肿瘤抑制方面有重要作用。

本文通过*Meta*分析，筛选出11个基因，其中*PTEN*、*PRKCB*及*CASK*三个基因在报道中与肺癌发病有重要关系，它们在抑制细胞增殖中有明显作用，与我们得到的基因处于下调通路结果一致。*PTEN*基因，即重组腺病毒第10号染色体同源丢失性磷酸酶张力蛋白基因（phosphatase and tensin homology delected on chromosome ten）是迄今发现的第一个编码具有磷酸酶活性蛋白质的抑癌基因，在调控细胞生长及细胞凋亡过程中起着重要作用^[5]^。*PTEN*基因的失活与多种肿瘤的发生发展密切相关, 在结直肠癌、乳腺癌、鼻咽癌、胃癌等多种癌组织中都已报道该基因的缺失或突变。目前有关其在肺癌中的表达改变研究近两年才有报道。舒红等^[11]^认为PTEN/PI3K/Akt信号途径可能参与了非小细胞肺癌的发生及恶性进展，而PTEN的表达情况可作为判断非小细胞肺癌预后的指标之一。

*PRKCB*基因蛋白激酶C（PKC）是基因家族的成员之一。*PRKCB*的基因功能涉及许多方面，比如，B细胞活化、诱导细胞凋亡、内皮细胞增殖以及肠道糖吸收。其中，B细胞活化以及诱导细胞凋亡功能提示*PRKCB*基因可能作为一抑癌基因在肺癌治疗中起重要作用。PRKCB抑制剂能够通过活化NF-κB信号通路而促使B细胞的死亡。此外在功能上，PRKCB可能与抗原受体介导的信号转换相关联。目前国内外鲜有其与肺癌相关报道。

诱导表达CASK可导致细胞周期依赖性激酶抑制因子等表达上调, 而它们发挥抑制细胞增殖功能的方式是导致细胞G_1_/S期阻滞，提示CASK可以通过调节细胞周期调控因子参与细胞周期的调控、抑制细胞的增殖功能。国内目前无与肺癌的相关报道。因为共同通路较多，我们首先选取最有意义的细胞通讯分类通路进行研究，后续我们将对这些差异显著基因进行验证。
